# Altered Functional Connectivity Between the Cerebellum and the Cortico-Striato-Thalamo-Cortical Circuit in Obsessive-Compulsive Disorder

**DOI:** 10.3389/fpsyt.2019.00522

**Published:** 2019-07-24

**Authors:** Haisan Zhang, Bi Wang, Kun Li, Xiaoyue Wang, Xianrui Li, Jianli Zhu, Qingjiang Zhao, Yongfeng Yang, Luxian Lv, Meng Zhang, Hongxing Zhang

**Affiliations:** ^1^The Second Affiliated Hospital of Xinxiang Medical University, Xinxiang, China; ^2^Xinxiang Key Laboratory of Multimodal Brain Imaging, Xinxiang, China; ^3^School of Psychology, Xinxiang Medical University, Xinxiang, China; ^4^Henan Key Lab of Biological Psychiatry of Xinxiang Medical University, Xinxiang, China; ^5^International Joint Research Laboratory for Psychiatry and Neuroscience of Henan, Xinxiang, China

**Keywords:** obsessive-compulsive disorder, cerebellum, cortico-striato-thalamo-cortical circuit, functional magnetic resonance imaging, FC

## Abstract

**Background:** Altered resting-state functional connectivity of the cerebellum in obsessive-compulsive disorder (OCD) has been previously reported. However, the previous study investigating cerebellar–cerebral functional connectivity relied on *a priori*–defined seeds from specific networks. In this study, we aimed to explore the connectivity alterations of the cerebellum in OCD under resting-state conditions with a hypothesis-free approach.

**Methods:** Thirty patients with OCD and 26 healthy controls (HCs) underwent functional magnetic resonance imaging (fMRI) scanning at resting state. Regional cerebral function was evaluated by measuring the fraction of amplitude of low-frequency fluctuation (fALFF). Regions with mean fALFF (mfALFF) alterations were used as seeds in seed correlation analysis (SCA). An independent samples t test was used to compare the differences in mfALFF and functional connection (FC) between the two groups. Pearson correlation analysis was performed to identify the association between functional neural correlates and OCD symptom severity evaluated using the Yale-Brown Obsessive Compulsive Scale (Y-BOCS).

**Results:** Compared with the HC group, the OCD group showed significantly increased mfALFF values in bilateral cerebellar. The results of FC analysis showed weakened connectivity among the left Crus II, lobule VIII, and right striatum and between the right lobule VIII and the right striatum, and cingulate in the OCD group compared with the HC group. Some of the abovementioned results were associated with symptom severity.

**Conclusions:** OCD patients showed abnormal spontaneous cerebellar activity and weakened functional connectivity between the cerebellum and the cortico-striato-thalamo-cortical (CSTC) circuit (striatum and cingulate), suggesting that the cerebellum may play an essential role in the pathophysiology of OCD.

## Introduction

Obsessive-compulsive disorder (OCD) is a common psychiatric condition, with a lifetime prevalence of 1% to 3% in the general population (1, 2). The core symptoms of OCD are obsessions and compulsions. OCD patients also experience deterioration of cognitive functions, such as attention, memory, decision-making ability, and inhibitory control (3, 4).

Multiple lines of evidence indicate that cortico-striato-thalamo-cortical (CSTC) circuit dysfunction is the core pathophysiological feature of OCD (5). In the CSTC circuit, the direct pathway projects from the prefrontal cortex to the striatum, then to the thalamus, and back to the prefrontal cortex (6, 7). Hyperactivity of the direct pathway is thought to lead to hyperactivation of the orbitofrontal cortex. Thus, excessive concern about danger, health, or injury mediated by the orbitofrontal cortex may lead to sustained conscious attention to perceived threats in OCD patients, leading to compulsive behavior aimed at eliminating perceived threats. The results of functional imaging studies support the above views. The most consistent findings in functional imaging studies of OCD pertain to abnormally increased activation of the lateral prefrontal cortex, including the orbitofrontal cortex and anterior cingulate (8, 9). Similarly, hyperactivity in the striatum and thalamus has been reported in OCD patients (10). However, recent studies have shown that OCD patients have structural and functional abnormalities in the cerebellum (11, 12), including increased cerebellar volume (12) and enhanced spontaneous cerebellar activity under resting state (13). The cerebellum is commonly associated with motor regulation, but recent experimental evidence suggests that it may also play a key role in cognition and emotion (14). Current evidence suggests that separate cerebellar regions are connected with different cerebral areas to form multiple cognitive circuits with topographic functions (15, 16). These functions include attention, language, working memory, visuospatial processing, and decision-making (14), which have been reported to be deficient in OCD patients (17, 18). This information is inevitably reminiscent of the role of cerebellar–cerebral functional connectivity in OCD.

The amplitude of low-frequency fluctuations (ALFFs) for the regional blood oxygen level-dependent (BOLD) signal, which is a method to investigate the regional spontaneous activity by calculating the square root of the power spectrum in the frequency range (19, 20), has been widely used in neuroimaging studies (21–23). The fractional ALFF (fALFF) approach (24) is an improved ALFF method, which measures the ratio of power spectrum of low-frequency range to that of the whole frequency range. Non-specific signal components could be effectively suppressed by this technology, and the sensitivity and specificity in examining regional spontaneous brain activity could be significantly improved. At present, this method has been successfully applied to the study of brain function (25–27). In addition, fALFF is also used in the selection of seed in functional connection analysis (28).

Seed correlation analysis (SCA) is a method of seed activation based on computed correlations with other areas of the brain that have similar temporal pathways, providing relatively precise and specific FC. This method has proven to be effective in revealing features of the brain connection network in psychiatric diseases and has been widely adopted (29, 30). In general, the seed can be selected based on a previous hypothesis or a collection of points based on prior fMRI studies. Currently, cerebellar–cerebral functional connectivity studies based on previous seed hypotheses have been reported, and they found that the cerebellum had reduced functional connections to a wide range of cortical regions, including parts of the frontal, temporal, occipital, and parietal lobes (31). However, there are still few studies on the cerebellar functional connections of OCD, and in view of the heterogeneity of experimental samples, a study with a hypothesis-free approach is necessary.

In the present study, we used a multiple-algorithm analysis in combination with fALFF and FC to explore the alterations in the resting-state functional connectivity of the cerebellum in OCD.

## Materials and Methods

### Subjects

Thirty right-handed adult patients with OCD were recruited from the Second Affiliated Hospital of Xinxiang Medical University. All patients were diagnosed by two psychiatrists according to the Diagnostic and Statistical Manual of Mental Disorders, 4th Edition (DSM-IV) criteria. Of the 30 OCD patients, 5 were drug-naïve and 25 were medication-free (patients who had discontinued medication for at least 2 weeks were considered medication-free); the medication-free patients mainly had received treatment with selective serotonin reuptake inhibitors, with the obsessive-compulsive symptom still existing or having relapsed when the cohort was recruited. None of the OCD patients had any other psychiatric disorder that met axis I of the DSM-IV. Twenty-six healthy controls (HCs) matched for age, gender, handedness, and education level were recruited as the control group. All HCs were screened using the Structured Clinical Interview for DSM-IV Axis I Disorders (SCID-I) to ensure that there was no history of other psychiatric disorders that met the criteria of axis I of the DSM-IV. The exclusion criteria for all participants included serious head injury, a history of drug or alcohol abuse, serious physical illness, and contraindications to MRI. All participants received a complete description of the study, and written informed consent was obtained. This study was approved by the Ethics Committee of the Second Affiliated Hospital of Xinxiang Medical University and complied with the Helsinki Declaration.

### Clinical Measures

OCD patients were assessed using the Yale-Brown Obsessive-Compulsive Scale (Y-BOCS), and all patients were required to have a Y-BOCS total score of ≥16.

### MRI Date Acquisition

Images were acquired with a 3 T MRI system (Siemens Magnet Verio) equipped with an eight-channel phased-array head coil. All subjects were told to relax and close their eyes during the scan. Foam padding and earplugs were used to reduce head motion and scanner noise. Resting-state BOLD images of the whole brain were acquired by using a gradient-echo echo-planar imaging sequence with the following parameters: time repetition (TR) = 2,000 ms, time echo (TE) = 30 ms, flip angle = 90°, matrix size = 64×64, field of view (FOV) = 220 mm, slices = 33, slice thickness = 4 mm, and time point = 240.

### Image Processing and Analysis

#### Date Processing

The preprocessing of the BOLD images was conducted using the Data Processing Assistant for Resting-State fMRI (DPARSF, http://rfmri.org/DPARSF), which is based on Statistical Parametric Mapping (SPM, https://www.fil.ion.ucl.ac.uk/spm/)and the toolbox for Data Processing & Analysis of Brain Imaging (DPABI, http://rfmri.org/DPABI). The images of the first 10 time points were removed to allow the signal to reach equilibrium. The remaining 230 points were corrected for slice timing and realigned for head motion correction. Subjects with more than 2 mm of maximal translation and 2° of maximal rotation were excluded. Previous studies have shown that a slight head movement can affect experimental results (32, 33). We also calculated framewise displacement (FD Jenkinson), which indexes the volume-to-volume changes in head position (34). There were no subjects with FD Jenkinson >0.2 mm, and there were no significant group differences in FD Jenkinson (*t* = 0.427, *p* = 0.675) between the OCD patients (0.057 ± 0.034) and controls (0.054 ± 0.027). Next, the motion-corrected functional volumes were spatially normalized to the MNI space and were resampled to 3 × 3 × 3 mm^3^ using the normalization parameters estimated during unified segmentation. The obtained images were smoothed with a 4-mm full-width at half-maximum (FWHM) Gaussian kernel. Further preprocessing included temporal bandpass filtering (0.01–0.08 Hz) and linear detrending.

### Statistical Analysis

The mfALFF was calculated using DPARSF. We transformed the time series into the frequency domain to obtain the power spectrum. After calculating the square root of each frequency in the power spectrum, we obtained the mean square root across a low-frequency range (0.01–0.08 Hz), which was regarded as the ALFF. fALFF is the ratio of the power of each frequency at the low-frequency range to that of the entire frequency range (24). Finally, the resulting spatial fALFF maps were then normalized with each voxel divided by the whole-brain fALFF mean, providing mfALFF spatial maps.

The mfALFF values of the whole brain of the two groups were compared with a two-sample t test using statistical parametric mapping SPM8. Although the age, gender, and years of education of the subjects in the two groups were matched, respectively, we still use them as covariables in the horizontal regression analysis of the group. Finally, we corrected statistical maps for Gaussian random field (GRF) correction to a significance level of *p* < 0.05, combining the voxel threshold *p* < 0.001 and cluster size >12 voxels.

The regions of cerebellar with changed mfALFF value were selected as seed regions for seed-based functional connectivity analysis using DPARSF. Temporary series of all voxels in each seed region were extracted and then averaged for each individual. Pearson correlation coefficients were computed between the seeds and the voxels of the whole brain to create the correlation maps for each seed and each participant. All correlation maps were transformed to z-value FC maps by applying Fisher’s r-to-z conversion (35) according to the equation:

$$z=\frac{1}{2}\ln\left(\frac{1+r}{1-r}\right)$$

where *r* is the Pearson correlation coefficient, and *z* is approximately a normal distribution.

Cerebellar–cerebral functional connectivity was compared between the two groups using two-sample t-test. The significance level was set at voxel *p* < 0.005 with GRF cluster correction of *p* < 0.05 (cluster size > 102 voxels).

The mfALFF values, z-fc values, and Y-BOCS scores of the OCD patients were analyzed by partial correlation analysis with age, gender, and years of education as the nuisance covariates. The significance level was set at *p* < 0.05/n after Bonferroni correction.

## Results

### Participants

After data collection, a total of 56 subjects were enrolled in the study, including 30 OCD patients and 26 controls. The demographic and clinical characteristics of the subjects are summarized in **Table 1**. There were no significant difference between the OCD group and the control group in age, gender, or years of education.

**Table 1:** Participant demographic and clinical features.

	OCD (n = 27)	HC (n = 25)	*t*/χ2	*p* Values
Age^a^	27.4 ± 8.9	27.8 ± 10.2	−0.173	0.864
Sex (M/F)^b^	14/16	10/16	0.383	0.596
Education, years^a^	11.0 ± 3.1	11.8 ± 2.5	−0.107	0.291
Y-BOCS	26 ± 5.9	N/A	N/A	N/A
Age of onset	20.77 ± 7.17	N/A	N/A	N/A
Duration of illness, years	6.88 ± 4.80	N/A	N/A	N/A

a Two independent sample t-test.

b Chi-square test.

### fALFF Analysis

**Figure 1:**
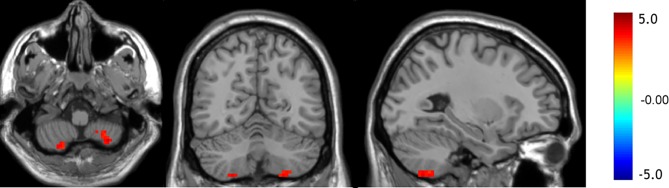
The regions showing increased mfALFF in OCD patients (*p* < 0.05, GRF corrected).

Compared to the control group, the values of mfALFF were increased in the left Crus II, the left lobule VIII, and the right lobule VIII of the cerebellum under resting state in the OCD group (see details in **Table 2** and **Figure 1**).

**Table 2:** The mfALFF value of cerebellum increased in OCD patients.

Brain regions	MNI coordinates			*t* values
	X	Y	Z	
Left Crus II	−11	−83	−40	5.392
Left Lobule VIII	−30	−63	−57	4.525
Right Lobule VIII	24	−69	−60	4.883

MNI, Montreal Neurological Institute.

### Seed-Based Functional Analysis

**Figure 2:**
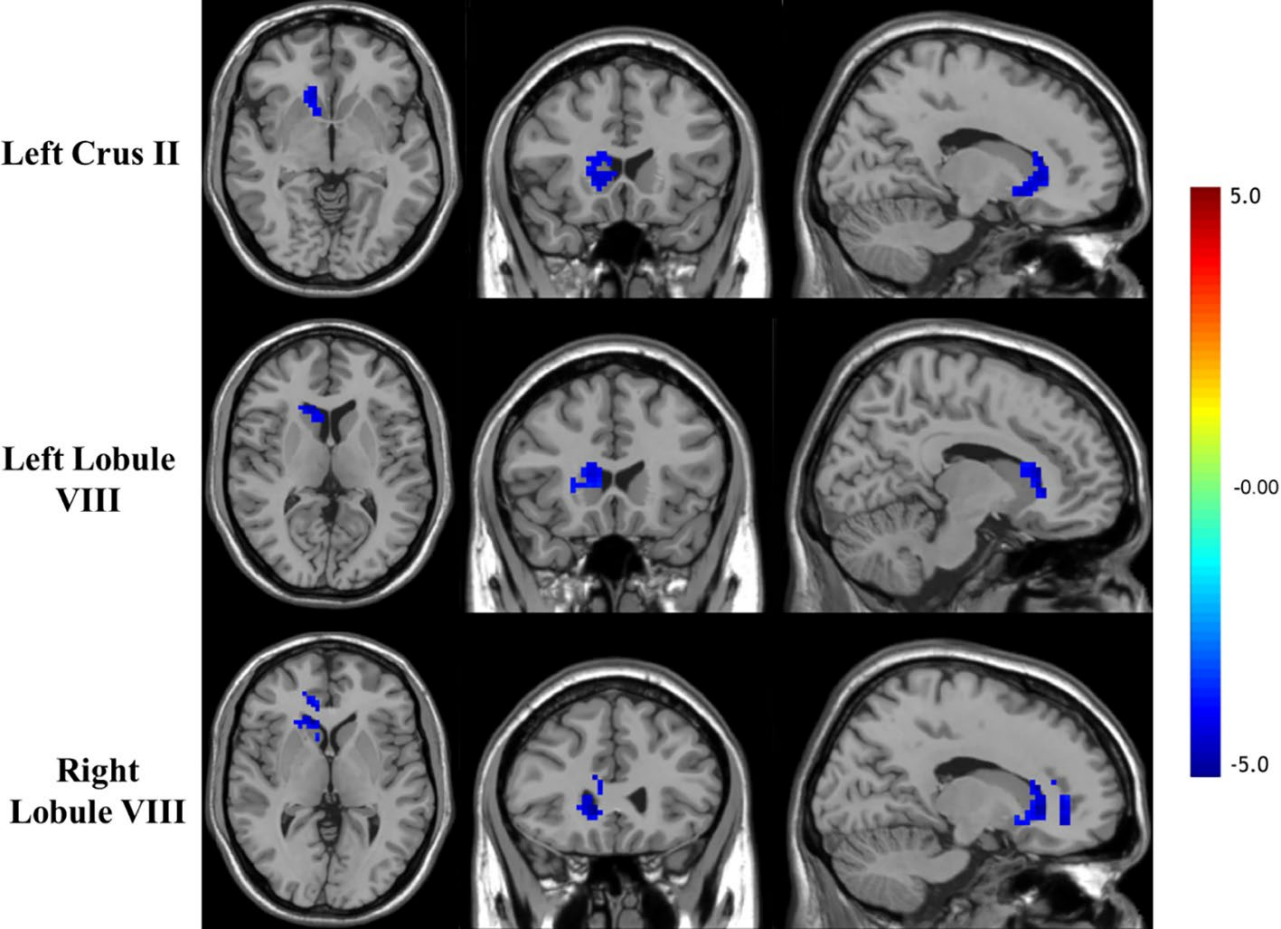
Brain regions showing decreased FC with the cerebellar seed in OCD patients (*p* < 0.05, GRF corrected).

mfALFF-difference regions of the cerebellum were selected as seeds for seed-based functional connectivity analysis. The results of FC analysis showed weakened connectivity among the left Crus II, lobule VIII, and right striatum and between the right lobule VIII, and the right striatum and cingulate in the OCD group compared with the HC group (see details in **Table 3** and **Figure 2**). Under the more rigorous correction (voxel threshold *p* < 0.001), there was no difference between the two groups.

**Table 3:** Brain regions showing decreased cerebellar–cerebral FC in the OCD group compared with the HC group.

**Brain regions**	**MNI coordinates**			***t*** ** values**
	**X**	**Y**	**Z**	
Left Crus II*				
Right striatum	18	24	12	−3.596
Left lobule VIII*				
Right striatum	15	24	12	−3.782
Right lobule VIII*				
Right striatum	12	24	0	−3.759
Right cingulate	12	41	0	−3.732
*Seed region.				

### Correlation Analysis

**Figure 3:**
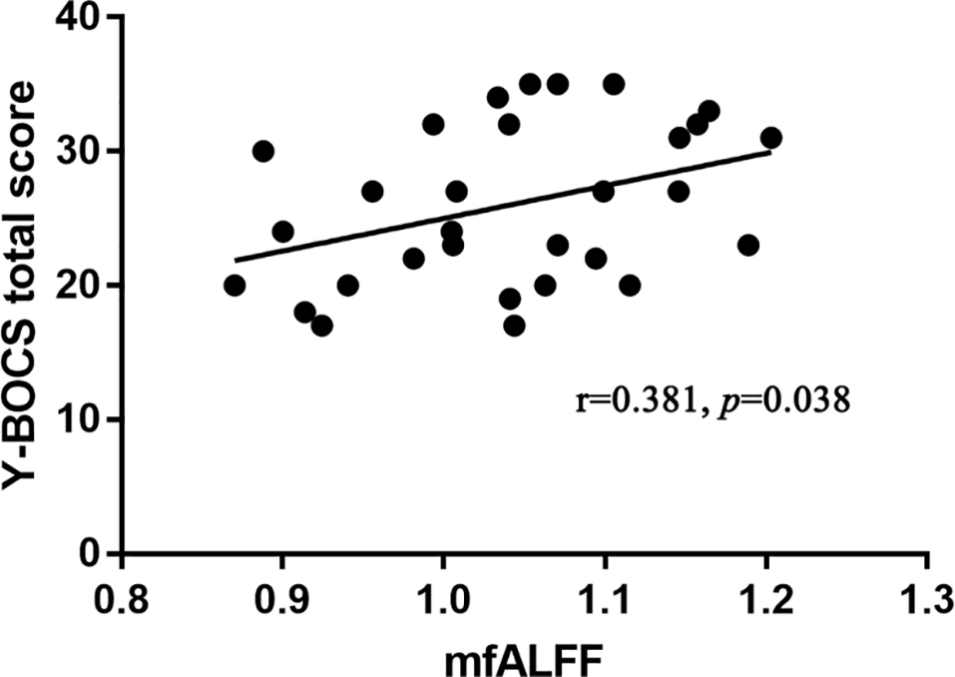
Significant positive correlation of mfALFF values in the left Crus II with the severity of obsessive-compulsive symptom in OCD patients (*p* < 0.05, Pearson correlation, uncorrected).

**Figure 4:**
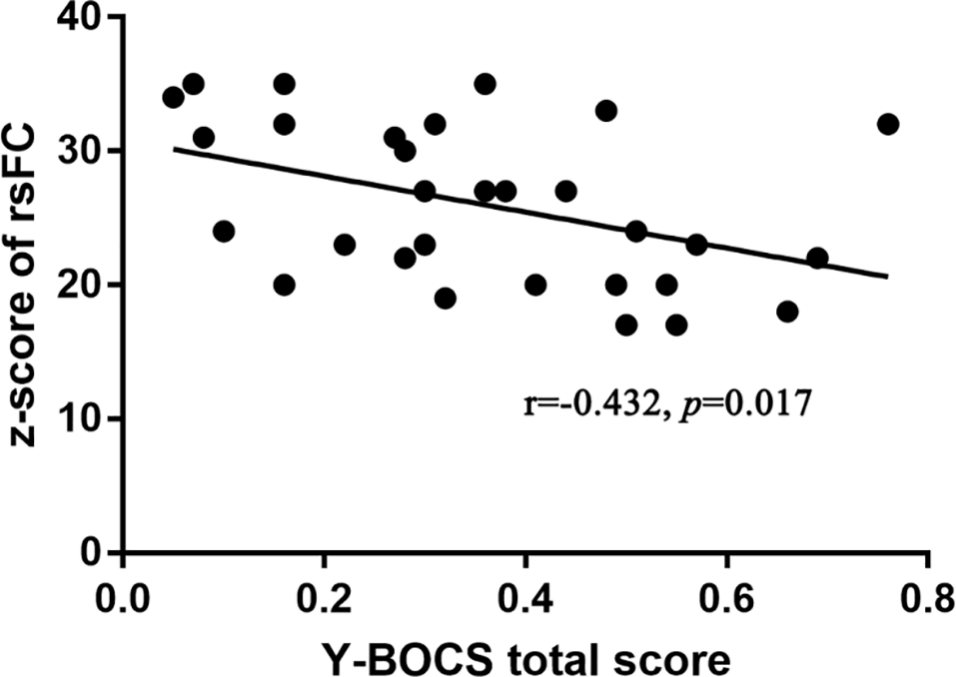
Significant positive correlation of FC between left Crus II and right striatum with the severity of obsessive-compulsive symptom in OCD patients (*p* < 0.05, Pearson correlation, Bonferroni corrected).

In OCD patients, the values of mfALFF in the right Crus II region of the cerebellum were positively correlated with the total score of Y-BOCS (see details in **Figure 3**). And after Bonferroni correction, the FC between the left Crus II and right striatum was negatively correlated with Y-BOCS total scores (see details in **Figure 4**).

## Discussion

In this study, we combined fALFF and FC to investigate the functional and connectivity alterations of the cerebellum in OCD. We found significantly increased mfALFF values in the bilateral posterior cerebellar lobes in the OCD patients, including the left Crus II, left lobule VIII, and right lobule VIII, consistent with the areas of the cerebellum that previous studies have suggested are involved in cognitive function. Furthermore, these cerebellar regions showed weakened functional connection with the striatum, suggesting that abnormal connection between the cerebellum and striatum may be involved in the neuropathology of OCD. In addition, some of the results above were associated with symptom severity.

### Cerebellum and OCD

Various studies have shown that people with OCD have an abnormal cerebellum presenting as abnormalities in structure, function, and blood flow (36–39). In addition, studies have also reported cases of OCD caused by cerebellar organic damage (40, 41). However, the focus on the role of the cerebellum in mental illness stems from its contribution to cognitive function. In motor regulation, the cerebellum is responsible for the expected forward control and error-based supervised learning in motor control. That is, the cerebellum, as a forward controller, predicts the consequences of the upcoming actions, compares and adjusts the errors with reality, and communicates the processed information to the corresponding cerebral cortex (42). Recent studies have shown that the cerebellum may regulate cognitive processes in a similar way, that is, as a forward controller to predict the consequences of cognitive activities and psychological expectations, then correcting its errors and conveying them to the cerebral cortex to help the cortex complete the correct cognitive activities (43). Interestingly, previous studies have shown that OCD patients have dysfunctional forward model mechanisms (44). In this study, the Crus II and lobule VIII regions we identified were in the posterior portion of the cerebellum and are involved in cognition (14), which to some extent confirmed this hypothesis. Previous studies have shown that the Crus II and lobule VIII regions are associated with executive function, whereas lobule VIII is involved in working memory (45, 46). Functional abnormalities in the above regions may be the cause of executive function and working memory deficits in OCD patients. However, some previous studies have reported different results. A recent rs-fMRI study showed decreased fALFF in the right cerebellum in treatment-naive OCD patients (47). In another study, female patients with OCD also had reduced fALFF in the cerebellum (48). The reasons for these differences may include sample heterogeneity, such as differences in patient gender composition and treatment status. Moreover, different data processing methods, such as differences in the FWHM during the smoothing process, may also be the cause of the difference in results. In addition, studies about the functional topography of the human cerebellum suggest that different regions of the cerebellum are involved in different functional domains, which may also be the cause of inconsistent results.

### Cerebellum and the CSTC Circuit

Earlier research may have been limited by the heterogeneity of OCD and the different research methods used. In previous studies, the results of abnormal cerebellar regions in OCD patients were not consistent, which caused difficulties in the selection of seed regions. A previous study investigating cerebellar–cerebral functional connectivity has relied on *a priori*–defined seeds from specific networks. In other words, seeds were selected based on different samples from the previous experiment. In view of the present evidence suggesting the heterogeneity of OCD (49), these seeds may not be suitable for different subjects. In this study, the fALFF method was first used to locate abnormal cerebellar regions in patients with OCD, and then the mfALFF value difference region was used as seed regions for whole-brain functional connectivity analysis. In this method, we did not make prior assumptions about the selection of seed regions, and this seed-based FC analysis approach may have advantages over studies based on *a priori*–defined seeds and provide more reasonable and persuasive findings regarding the pathogenesis of OCD. We found that the cerebellar regions with increased activity in OCD patients had decreased functional connectivity with the components in the CSTC circuit, including the striatum and cingulate. Additionally, decreased functional connectivity between the left Crus II and right striatum was negatively correlated with the severity of obsessive-compulsive symptoms. This is different from previous findings by Xu et al. (31). Differences in the above research methods may account for this result. Interestingly, our findings are more relevant for correlating the cerebellum with the CSTC circuit. Recent research shows that the cerebellum has extensive communication with regions in the CSTC circuit, such as the striatum and thalamus, and the communication was associated with nonmotor function (50–52). The striatum has always been suggested to be involved in habit formation and goal-directed action, and striatum dysfunction is an important cause of OCD (53–55). Alterations of the striatum have also been reported in both structural and functional neuroimaging studies in OCD (12, 56). Previous studies have shown bidirectional anatomical and functional connections between the cerebellum and the striatum (52, 57), and the abnormal functional connections may lead to some cognitive impairment, such as in reward and executive control (58–60). We hypothesized that decreased functional connectivity between the cerebellum and the striatum might affect striatum function, resulting in the inability of the striatum to effectively inhibit the thalamus. The hyperactive thalamus then leads to excessive activation of the frontal cortex, causing dysfunction in executive control and reward.

The cingulate is also an important part of the CSTC circuit, which is mainly related to activity inhibition, decision-making, and emotion regulation (61). Other studies have shown that the cingulate gyrus is closely related to attention (62). In previous studies, OCD patients showed decreased gray matter (GM) and hyperactivity in the cingulate (63–65). Due to the weakened connection between the cerebellum and cingulate, OCD patients may not be able to effectively transmit feedforward information to the anterior cingulate cortex, which makes it difficult for OCD patients to effectively inhibit forced movements and transfer their attention from compulsive thoughts, behaviors, or fears.

However, there are still some limitations in this study. On the one hand, the sample size of this study was small. On the other hand, we did not find any abnormal connections between brain regions and seed regions in OCD patients under a more rigorous correction (voxel threshold *p* < 0.001). The reason may be the lack of statistical effectiveness due to the small sample size. This study also lacked direct evidence regarding the relationship between abnormal neural correlates and cognitive deficits in OCD patients. We also did not perform a longitudinal comparison before and after treatment. Therefore, we still need supplementary information in future studies to provide further evidence for the role of the cerebellum in the pathogenesis of OCD.

## Conclusion

In summary, this study demonstrated abnormal spontaneous cerebellar activity and weakened functional connectivity between the cerebellum and the CSTC circuit in OCD patients, and some of the abovementioned results were associated with symptom severity. Our findings provide additional evidence that the cerebellum may play an essential role, and alteration of functional connections between the cerebellum and the CSTC circuit may be involved in the pathophysiology of OCD.

## Ethics Statement

In this study, all participants received a complete description of the study and written informed consent was obtained. This study was approved by the Ethics Committee of the Second Affiliated Hospital of Xinxiang Medical University, and complied with the Helsinki Declaration.

## Author Contributions

HZ and BW were involved in data collection, analysis, interpretation of results, and writing of the manuscript. KL was involved in data collection and preprocessing of the data. JZ, XL, QZ, YY, and XW were involved in subject recruitment and data collection. LL, MZ, and HZ were involved in study conception, design, manuscript editing, and intellectual content, and supervised all aspects of the study.

## Funding

The research was supported by NSFC-Henan mutual funds (U1704190), the National Nature Science Foundation of China (31600927, 81071091, 81671330, and 81571315), Innovation Scientists and Technicians Troop Construction Projects of Henan Province (174100510024), the Program for Innovative Research Team in Science and Technology in University of Henan Province (18IRTSTHN025), the Open Program of the Institute of Mental (2016PN-KFKT-10 and 2016PN-KFKT-28), the Open Program of the Henan Biological Psychiatry Key Laboratory (ZDSYS2014006 and ZDSYS2015002), High Scientific and Technological Research Fund of Xinxiang Medical University (2017ZDCG-04), Henan Science and Technology Program foundation and frontier project (162300410246), and the Natural Science Research Plan of the Department of Education of Henan province (12A320018).

## Conflict of Interest Statement

The authors declare that the research was conducted in the absence of any commercial or financial relationships that could be construed as a potential conflict of interest.
